# Prevalence of Functional Constipation in Children with Down Syndrome: A Study Conducted at a General Pediatrics Service

**DOI:** 10.3390/biomedicines14010162

**Published:** 2026-01-12

**Authors:** Ana Maria Daun Cação Pereira, Catarina Shin, Ana Beatriz Henrique Parenti, Mary de Assis Carvalho, Cátia Regina Branco Fonseca

**Affiliations:** Department of Pediatrics, Botucatu Medical School, São Paulo State University (UNESP), Botucatu 18618-687, Brazil; ana-maria.pereira@unesp.br (A.M.D.C.P.); catarina.shin@unesp.br (C.S.); a.parenti@unesp.br (A.B.H.P.); mary.carvalho@unesp.br (M.d.A.C.)

**Keywords:** Down Syndrome, infant, child, constipation, gastrointestinal disorders, functional

## Abstract

**Background/Objectives:** Gastrointestinal and eating disorders are highly prevalent problems in children with Down syndrome (DS) and have a significant impact on their daily lives. It is important to investigate the bowel habits of children with DS, specifically the prevalence of functional constipation (FC), in order to develop practice guidelines for pediatricians that support diagnosis and treatment. **Materials and Methods:** This observational, analytical, and cross-sectional study was approved by the Ethics Committee and included 36 children with DS under pediatric outpatient follow-up at a university hospital. To assess bowel habits, an interview was held with the parents using the Rome IV criteria and the Bristol Stool Scale. The children were divided into two groups: those with and those without FC. Specific curves for individuals with DS were used for nutritional assessment. **Results:** The median age of the children was 46.6 months (5 to 144 months); a total of 80.5% of those included were eutrophic. The median age at initiation of toilet training was 36 months. Most children achieved bowel control when training started after 30 months of age. A total of 15 (41.7%) of the 36 children included were assigned to the group with FC, and 21 (58.3%) were assigned to the group without FC. The FC group had a lower frequency of bowel movements, hardened stools, pain during bowel movement, and used laxatives. According to the Rome IV criteria, the three most prevalent criteria were hard stools, large-volume stools, and painful bowel movements. **Conclusions:** Children with DS had a high prevalence of FC, and it was possible to identify an association between delayed sphincter training and FC. A care and monitoring protocol and flowchart are useful tools for the general pediatrician.

## 1. Introduction

Down syndrome (DS) is the most prevalent chromosomal abnormality in the world [1], with an estimated incidence of 1 in 715 live births that increases with maternal age. The disease occurs independently of social class, ethnicity, or gender and is the most common cause of intellectual disability in the population [2,3,4]. In Brazil, statistical data on the incidence of newborns with DS remain unclear, with studies reporting an incidence ranging from 1 in every 600 to 700 births to 1 in every 2500 births [1,5,6].

Children with DS are predisposed to developing various comorbidities that interfere with their growth and development due to the presence of extra genetic material from chromosome 21 [7]. The condition is characterized by a higher incidence of different health conditions that affect their quality of life, including prematurity, congenital heart diseases, malformations, and thyroid function disorders, among others [2,3,8]. Gastrointestinal and eating disorders are highly prevalent problems in children with DS and have a significant impact on their daily lives [9]. 

The range of gastrointestinal alterations found in children with DS is associated with a high risk of developing chronic constipation. Constipation, which is defined as a set of symptoms of multifactorial causes, is a frequent functional gastrointestinal disorder in this population. It is a chronic or recurrent gastrointestinal condition characterized by the elimination with effort of feces of hard consistency, often accompanied by a reduction in the number of bowel movements per week and changes in fecal characteristics. There is a variable combination of symptoms that can compromise the quality of life of children with DS and can also interfere with their growth and development [9].

Constipation is defined as functional constipation (FC) if there is no underlying organic cause, which is the case in up to 95% of children. In the 5% with organic cause, the etiology varies from Hirschsprung disease, anorectal malformations, neuromuscular disease, and metabolic issues to endocrine disorders [10]. 

Functional constipation is a common disorder in all age groups, but the peak incidence of constipation occurs between 2 and 4 years of age, when toilet training (TT) starts. Recent studies found that the median prevalence of constipation in children was 12% [10,11].

The pathophysiology of FC in children remains unclear but is multifactorial. The most common, especially in young children, is withholding behavior, often starting after a painful, frightening bowel movement [12]. The stools remain in the rectum, and the rectal mucosa reabsorbs water from the retained stools, which become more difficult to evacuate. This vicious circle can lead to fecal impaction, sometimes to overflow fecal incontinence, loss of rectal sensation, and ultimately, loss of the normal urge to defecate [13].

The diagnosis of FC is according to the Rome IV criteria and the Bristol scale. The Rome IV criteria for younger children, the age group 0–4 years of age, were considered. Children who are not trained in toilets do not need to fulfill two additional criteria to be diagnosed with FC. Rome IV criteria are proposed as the new gold standard; they should be evaluated like all other definitions [14].

The Rome IV criteria, compared to Rome III, eliminate two diagnostic criteria in children under the age of four who still wear diapers. Physical examination and taking a thorough medical history are recommended, but other investigations such as abdominal radiography, transabdominal recto-ultrasonography, colonic transit time, rectal biopsies, and colon manometry are not routinely recommended [14].

The criteria include the following: Duration of symptoms (must include at least 1 month); at least two or more criteria; diagnosis criteria, two or fewer defecations/week; history of painful or hard bowel movements; and a large fecal mass in the rectum. And there are differences depending on whether the child’s age is less than or more than four years: excessive stool retention; toilet-trained children have additional criteria; large-diameter stools or mass; and at least one episode of fecal incontinence/week or large-diameter stools that could obstruct the toilet [14]. 

The recommendation for the management of FC includes a normal intake of fibers and fluids, normal physical activity, and an additional pharmacologic treatment for fecal disimpaction followed by a pharmacologic maintenance therapy [10,14].

The present study is justified by the need to increase pediatricians’ knowledge about bowel habits and TT in infants and children with DS, to identify the prevalence of FC diagnosed based on the Rome IV criteria, and to develop a protocol for pediatricians that addresses FC in DS that helps improve the growth and development of these children and optimizes the quality of life of both patients and their parents/caregivers. Therefore, the aim of this study was to evaluate the prevalence of FC in children with DS undergoing regular follow-up at a general pediatrics service of a university, as well as to assess bowel habits and the characteristics of TT.

## 2. Materials and Methods

This was an observational, cross-sectional, and descriptive clinical study that used interviews and standardized questionnaires to investigate bowel habits in infants and children undergoing outpatient pediatric follow-up at the University Hospital of the Botucatu Medical School (HCFMB). 

It is important to note that this clinic is a general pediatric clinic, not a specialized pediatric gastroenterology or pediatric genetics service, which explains why the number of participants included in the study is not so large, but is nonetheless representative of the reality of a general pediatric service.

### 2.1. Eligibility Criteria and Sample Size

Children diagnosed with DS who were followed up with at the Childcare and Pediatrics outpatient clinics of HCFMB were included. All children included in this convenience sample periodically underwent laboratory tests following the recommendations of the Brazilian Society of Pediatrics [2]. The protocol for data collection was applied during the routine consultations of these children.

The inclusion criteria were age of 30 days to 12 years, a confirmed diagnosis of DS by karyotyping, and consent of the family member for participation in the study.

For the diagnosis of intestinal constipation, the Rome IV criteria (Box 1) and the Bristol Stool Scale (Figure S1) were used, which were translated and adapted for the Brazilian population [15].

Box 1 **Rome IV criteria for constipation.**

At least 2 of the following should be present for at least 2 months:

1. Two or fewer defecations per week.
2. At least 1 episode per week of incontinence after the acquisition of toileting abilities.
3. History of excessive stool retention.
4. History of painful or hard bowel movements.
5. Presence of a large fecal mass in the rectum.
6. History of large-diameter stools that may obstruct the toilet.
     Rome IV criteria for children and adolescents

Two or more of the following for a child aged at least 4 years with sufficient criteria for at least 2 months:

1. Two or fewer defecations per week.
2. At least 1 episode of fecal incontinence per week.
3. History of retentive posturing or excessive volitional stool retention.
4. History of painful or hard bowel movements.
5. Presence of a large fecal mass in the rectum.
6. History of large-diameter stools that may obstruct the toilet.

Source: Adapted from Rasquin et al., 2016; Rome Foundation [16].

Children with organic gastrointestinal disorders (Hirschsprung’s disease, celiac disease, and imperforate anus) were excluded. 

All children with thyroid dysfunction (hypothyroidism or hyperthyroidism) were considered and included in the group if they had received adequate treatment and had normal laboratory test results (TSH and free T4) at the time of their inclusion in the study. 

### 2.2. Clinical Evaluations

Anthropometric assessment of the weight and height of patients with DS was performed immediately before the medical consultation. The AnthroCalc^®^ (World Health Organization, Geneva, Switzerland), application was used to calculate weight-for-age, height-for-age, and weight-for-height z-scores (for children <2 years), body mass index (BMI), and BMI-for-age z-scores (for children ≥2 years). The scores were classified as proposed by Zemel et al. [17] for children with DS. The cutoff values recommended by the World Health Organization were used to define nutritional status based on z-scores [18].

Data were collected using a clinical protocol specifically developed for the study, which was applied during the routine medical consultation of the children and answered by the caregiver. The protocol included questions regarding the Rome IV criteria and Bristol Stool Scale (Figure S1) [15] and a protocol for assessing TT [19] based on the readiness signs of Brazelton [20]. Sociodemographic and clinical variables on bowel habits were also collected.

The age at initiation of TT was defined as the age at which parents/caregivers introduced the potty or toilet and started to discuss training aspects with the child. Achieving full bowel and bladder control (complete TT) was defined when parents/caregivers reported that the child no longer wore diapers and used the potty or toilet regularly. Signs of readiness for initiating TT shown by infants and children with DS who achieved bowel control were also considered.

### 2.3. Statistical Analysis

The database was prepared in a Microsoft Excel ^®^, Microsoft 365 (Microsoft Corporation, Redmond, WA, USA) spreadsheet and analyzed using the GraphPad Prism^®^, version 9.0 for Windows (GraphPad Software, San Diego, CA, USA). For the definition of parametric or nonparametric tests, the Kolmogorov–Smirnov test was applied to determine whether the variables had a normal distribution. For descriptive statistics, measures of central tendency were used (simple frequency, relative frequency, mean and standard deviation, and median and interquartile range). Qualitative variables were compared using Fisher’s exact test. The *t*-test and Mann–Whitney test were used to compare quantitative variables [21].

A binomial regression analysis was performed to evaluate the factors associated with CF, as shown in the results.

## 3. Results

Thirty-six children with DS, ranging in age from 5 months to 12 years, were included and divided into two groups according to the Rome IV diagnostic criteria: children with and without FC. Of these, 15 (41.7%) were assigned to the group with FC and 21 (58.3%) to the group without FC.

Seven of the children in the study had hypothyroidism controlled by medication; of these, only one was included in the constipation group. Two adolescents had hyperthyroidism, also controlled, with one in each study group.

Mothers were the main caregivers (94%) who responded to the protocols. There were no statistically significant differences between the groups with and without FC for sociodemographic variables such as age or education level of the parents or household composition (average of four household members, homes with few rooms, and low occupancy) (Table 1).

One-third of the participants did not have comorbidities. Among participants with comorbidities, congenital heart disease was the most prevalent (n = 10, four in the FC group), with a predominance of interatrial communication. The other frequent comorbidities were congenital hypothyroidism (n = 7, one in the FC group), gastroesophageal reflux disease (n = 5, four in the FC group), hyperthyroidism (n = 2, one in the FC group), epilepsy (n = 2, one in the FC group), and Dandy–Walker syndrome and esophageal atresia, with one case each in the group without FC. In the group with FC, there was one child with hypothyroidism undergoing treatment. This child was not excluded from the sample of the FC group because he was already being treated and had normal thyroid hormone levels.

Table 2 shows the nutritional status of the children; a total of 93.3% in the group with FC and 71.4% in the group without FC were eutrophic.

Infants and children with DS who achieved bowel control (complete TT) showed the following 11 signs of readiness for initiating TT: walking (81.3%), knowing how to say “no” (81.3%), talking (75%), having words for poop or pee (75%), imitating parents’ behavior (75%), already understanding and executing a simple order (68.8%), being able to sit and concentrate on an activity (56.3%), complaining when dirty (56.3%), already knowing where to put something (50%), evacuating at scheduled times (50%), and putting on and taking off clothes alone (50%), as shown in Table 3.

Regarding the characteristics of bowel movements of infants and children with DS included in the study, most participants had daily bowel movements, formed or pasty, without pain or difficulty evacuating and without fecal leakage. However, the percentages of decreased bowel movement frequency, hard stools, pain during evacuation, and use of laxatives were significantly higher in the group with FC compared to the group without FC. As can be seen in Table 4, the three most prevalent diagnostic criteria of the Rome IV criteria among participants with FC were hard stools, large-volume stools, and painful bowel movements.

Using binary logistic regression, only delayed toilet training remained significantly associated with functional constipation in children with Down syndrome. After adjusting for age, children with delayed acquisition of bowel control showed a substantially higher likelihood of presenting functional constipation (OR = 9.77; 95% CI: 1.20–80.01; *p* = 0.034). No other variables included in the model demonstrated significant associations with the outcome (Table 5).

At the end of the study, a protocol for investigating bowel habits in children with DS was developed to support pediatricians based on a flowchart for assessing and indicating imaging tests and/or referral to specialists, such as pediatric gastroenterologists, who can better manage complex cases (Figure S2).

## 4. Discussion

In the present study, the prevalence of FC according to the Rome IV criteria was much higher among children with DS compared to the population of children without DS, both in Brazil and worldwide. According to meta-analysis, the combined global prevalence of FC in children without syndromes is 9.5% (95% CI, 7.5–12.1%), ranging from 0.5% to 32.2% [22]. 

In Brazil, two recent studies applying the Rome III criteria reported a prevalence of FC of 10.4% and 10.9% among non-syndromic children aged 5 to 17 years [23,24]. In our study, the prevalence of FC among children with DS was approximately four times higher. This increased prevalence of FC was similar to that reported by Ciciora et al. [25], who found a prevalence of 36% among American children with DS who met the Rome IV criteria. A study conducted in Brazil on children with DS reported similar results, with a prevalence of 49% [26]. On the other hand, our results differed from those reported by Skotko et al., who found a prevalence of FC of 18% among American children with DS also treated at a tertiary service [27].

The high prevalence of FC can be explained by variations in the enteric nervous system (ENS) demonstrated in human and murine models of DS, as well as alterations in the central nervous system (CNS), with both playing a key role in functional gastrointestinal disorders. The hypothesis is that the alterations related to neurotropic factors that occur during embryogenesis of the CNS in patients with DS would also occur in the ENS [28], leading to functional or organic disorders. Furthermore, hypotonia of the abdominal muscles in children with DS might be an additional factor that would explain the increased rates of FC [25]. This condition is considered a potential organic source of constipation, especially in younger children in whom hypotonia is more pronounced [29]. Thus, given the medical complexities of individuals with DS, the possibility of other hidden organic diagnoses mimicking a functional disorder cannot be ruled out. However, previous studies have shown that organic diseases rarely meet the diagnostic criteria of a functional disorder, a fact that renders the possibility of an incorrect functional diagnosis less likely [25,30,31].

The sociodemographic, anthropometric, and nutritional characteristics, as well as the prevalence of comorbidities, were similar between the groups with and without FC, demonstrating the homogeneity of the groups evaluated. In our study, no differences were found between the two groups in terms of the presence of hypothyroidism, which is also observed by Ciciora et al., whose patients with DS and hypothyroidism did not have an increased risk of FC [25].

Toilet training is an important developmental milestone that enables children to achieve greater autonomy and access to academic and social opportunities [19,32]. Children with DS are a group that is more vulnerable to the challenges of TT. In our study, we found a delay in the initiation of TT in children with DS, which occurred in most cases before the 4th year of life. The duration of TT until achieving full bowel and bladder control was short (6.5 months). Bowel control was achieved at a median age of 3.5 years, but was the most delayed, which led to a delay in attaining complete TT. These data are consistent with studies involving this population [19,32]. Despite the delay in initiating and completing TT observed in our sample, the duration of training was shorter than that reported in previous studies and occurred relatively early when compared to studies conducted in other countries [19,32].

These differences between studies are probably due to cultural and economic factors of the populations studied. Mothers with a lower education level and concerns regarding diaper costs tend to start training earlier [33]. Increasing the chances of early access to school is another reason for early initiation of TT [32]. Another important factor is caregiver availability for TT. In our sample, the main people responsible for TT were the parents themselves (68%), and training was performed in their homes.

The child-centered approach was the most frequently used method for initiating TT, which was guided by the characteristic signs of readiness described by Brazelton, which are in line with the studies by Dreher et al. and Mrad et al. [19,32]. This TT model considers three forces of child development: physiological maturation (e.g., the ability to sit, walk, dress, and undress); external feedback (e.g., understanding and responding to instructions), and internal feedback (e.g., self-esteem and motivation, desire to imitate and identify with mentors, self-determination, and independence) [20,22].

An association between delayed toilet training and functional constipation was observed in our study, which differs from the findings of Mrad et al., who did not identify such an association. This discrepancy may be explained by methodological differences, including the use of the Rome III criteria in the previous study, which are less sensitive than the Rome IV criteria, as well as differences in sample composition. Thus, variations in diagnostic criteria and sample characteristics may account for the divergent findings between the studies [32].

One limitation of the present study was the small number of patients and the wide variation in age within groups. This was a single-center study conducted at a tertiary service, using a convenience sample, with the data collection period coinciding with the COVID-19 pandemic, which resulted in a reduction in patient visits during that time. It is important to note that this clinic is a general pediatric clinic, not a specialized pediatric gastroenterology or pediatric genetics service. Nevertheless, the results were useful to understand the high prevalence of FC in DS since the study used a systematic approach to patients based on interviews and self-completed questionnaires by the caregivers under the supervision of a pediatric gastroenterologist, who was the main researcher.

## 5. Conclusions

In conclusion, the prevalence of CF in children with DS was high, as was that of delayed bowel control, and it was possible to identify an association between delayed sphincter training and functional constipation. The appropriate diagnostic and therapeutic approach for children and adolescents with DS, as proposed in the flowchart presented, should assist general pediatricians in providing adequate care to this population.

## Figures and Tables

**Table 1:** Sociodemographic characteristics of parents and their children with Down syndrome.

Characteristics	Total (n = 36)	With FC (n = 15)	Without FC (n = 21)
	Median (IQR) or n (%)		
Child			
Age (months)	46.6 (14.3–91.2)	33.3 (14.1–95.4)	48.9 (19.9–90.3)
Female sex	16 (44.4)	9 (60.0)	8 (38.0)
Parents			
Maternal Age (Years)	40.5 (35.2–46)	38 (35–44)	41 (35.2–47.7)
Paternal Age (Years)	42 (35.2–49.5)	42 (34–53)	41 (35.5–47)
Maternal Education			
Elementary School	15 (41.6)	4 (26.6)	9 (42.8)
High School	15 (41.6)	9 (60)	8 (38.1)
Higher Education	6 (16.6)	2 (13.3)	4 (19.1)
Paternal Education			
Elementary School	12 (33.3)	3 (20)	9 (42.8)
High School	17 (47.2)	9 (60)	8 (38.1)
Higher education	7 (11.1)	3 (20)	4 (19.1)
Caregiver/Respondent			
Mother	34 (94)	14 (93.3)	20 (95.2)
Father	2 (6)	1 (6.7)	1 (4.8)
Household Composition			
Number of Rooms	5 (4–7)	5 (4.7–6.2)	5 (4–7)
Number of Household Members	4 (3–4.7)	4 (3–5)	4 (3–4)
Number of Children	2 (1–2)	2 (1–3)	2 (1–2)
Number of Adults	2 (2–2)	2 (2–2)	2 (2–2)
Occupancy (Persons/Room)	0.7 (0.5–1)	0.8 (0.6–1.2)	0.6 (0.5–1)
Urban Area	33 (91.6)	14 (93.3)	19 (90.4)

FC, functional constipation; IQR, interquartile range. Comparison between groups with and without constipation by the Mann–Whitney test. There were no statistically significant differences for any of the variables evaluated (*p* > 0.05).

**Table 2:** Anthropometric characteristics of children with Down syndrome.

Characteristics	Total (n = 36)	With FC (n = 15)	Without FC (n = 21)
	Median (IQR) or n (%)		
Age at Consultation (Months)	46.6 (14.3–91.2)	33.3 (14.1–95.4)	48.9 (19.9–90.3)
**Nutritional Status**			
Weight-for-Age z Score	−0.12 (−0.89–0.67)	−0.14 (−1.28–0.3)	−0.11 (−0.76–0.95)
Height-for-Age z Score	0.48 (−0.36–0.95)	0.53 (−1.86–0.82)	0.44 (−0.34–0.96)
Weight-for-Height z Score (<2 years)	−0.73 (−1.79– −0.14)	−0.21 (−0.96–0.69)	−1.5 (−2.77– −0.73)
BMI-for-Age z Score (≥2 years)	−0.27 (−1.19–0.8)	−0.48 (−1.25–0.52)	0.2 (−0.96–0.92)
Eutrophic, n (%)	29 (80.5)	14 (93.3)	15 (71.4)
Malnourished, n (%)	4 (11.1)	1 (6.7)	3 (14.3)
Obese/overweight, n (%)	3 (8.3)	0	3 (14.3)

FC, functional constipation; IQR, interquartile range; BMI, body mass index. Comparison between groups with and without constipation by the Mann–Whitney test and Fisher’s exact test. There were no statistically significant differences for any of the variables evaluated (*p* > 0.05).

**Table 3:** Characteristics of toilet training of children with Down syndrome who achieved bowel control.

Characteristics	Children with Bowel Control (n = 16)
	Median (IQR) or n (%)
Age at Consultation (Months)	90.3 (56.8–135.9)
Female sex	9 (56.2)
Age at Initiation of Toilet Training (Months)	36 (33–48)
<18	0
18–30	3 (18.8)
>30	13 (81.2)
Age at Achieving Bowel Control (Months)	42.5 (38.2–54.5)
<48	9 (56.3)
>48	7 (43.7)
Total Duration of Toilet Training (Months)	6.5 (5.5–11)
Person Responsible for Toilet Training	
Mother	5 (31.2)
Mother and Father	4 (25)
Mother, Father, and Teachers	3 (18.8)
Father	2 (12.5)
Teachers	1 (6.2)
Place of Toilet Training	
Parents Home	11 (68.7)
Parents’ Home and Daycare/School	4 (25)
Only Daycare	1 (6.2)
Bowel and Bladder Training	
Simultaneous	13 (81.2)
Not Simultaneous	3 (18.8)
First Control, Bowel or Bladder	
Simultaneous	13 (81.2)
Bladder	4 (25)
Bowel	3 (18.8)
Device Used for Toilet Training	
Toilet	9 (56.2)
Potty	6 (37.5)
Other (bathtub)	1 (6.2)
Source of Training Knowledge	
Medical Advice	6 (37.5)
Experience with Previous Child	5 (31.2)
Intuition	3 (18.8
Other	1 (6.2)
Pressure to Remove Diaper	
No Pressure	12 (75)
School	2 (18.8)
Relatives	1 (6.2)
Cost of Diapers	0
Maternal Attitude Towards the Child’s Refusal to Train	
Counseling	16 (100)
Reasons for the Child’s Refusal to Accept Training	
Fear of Sitting on the Toilet	4 (25)
Fear of Sitting on the Potty	0

IQR, interquartile range.

**Table 4:** Characteristics of bowel habits of children with DS according to the Rome IV criteria.

	Total (n = 36)	With FC (n = 15)	Without FC (n = 21)	*p*-Value *
	Median (IQR) or n (%)			
Age at Consultation (Months)	46.6 (14.3–91.2)	33.3 (14.1–95.4)	48.9 (19.9–90.3)	0.58
Characteristics of Bowel Habits				
Frequency of Bowel Movements				
Daily	29 (80.6)	9 (60)	20 (95.2)	<0.001
3–4xx/week	5 (13.9)	4 (26.7)	1 (4.8)	
1–2xx/week	2 (5.6)	2 (13.3)	0 (0)	
<1xx/week	0 (0)	0 (0)	0 (0)	
Pain or Difficulty Evacuating				
No Difficulty	24 (66.7)	7 (46.7)	17 (81)	0.001
Occasional	6 (16.7)	2 (13.3)	4 (19)	
Frequent	0 (0)	0 (0)	0 (0)	
During Every Evacuation	6 (16.7)	6 (40)	0 (0)	
Stool Consistency				
Formed or Pasty	26 (72.2)	7 (46.7)	19 (90.5)	<0.001
Hard	6 (16.7)	4 (26.7)	2 (9.5)	
Large and/or Scaly	3 (8.3)	3 (20)	0 (0)	
Very Large and Hard	1 (2.8)	1 (6.7)	0 (0)	
Fecal Leakage				
Never	29 (80.6)	10 (66.7)	19 (90.5)	0.18
1xx/week	3 (8.3)	3 (20)	0 (0)	
>1xx/week	3 (8.3)	2 (13.3)	1 (4.8)	
Every Day	1 (2.8)	6 (40)	1 (4.8)	
Use of Bathroom				
To Evacuate Alone	9 (25)	3 (20)	6 (28.6)	0.62
To Evacuate Alone or When Reminded	5 (13.9)	2 (13.3)	3 (14.3)	
Only When Reminded	0	0 (0)	0 (0)	
Does Not Use the Bathroom	22 (61.1)	10 (66.7)	12 (57.1)	
Use of Laxatives				
Does Not Use	27 (75)	6 (40)	21 (100)	<0.0001
Irregular Use	1 (2.8)	1 (6.7)	0 (0)	
Regular Use	8 (22.2)	8 (53.3)	0 (0)	
Use of Enemas for Bowel Cleansing	0	0 (0)	0 (0)	

IQR, interquartile range. * Comparison between groups with and without constipation by the Mann–Whitney test and Fisher’s exact test.

**Table 5:** Factors associated with functional constipation in children with DS (n = 36).

Variable	Category	Without FC n (%)	With FC n (%)	*p*-Value
Sex	Male	13 (61.9%)	6 (40.0%)	^β^ 0.194
	Female	8 (38.1%)	9 (60.0%)	
Thyroid Disorders	No	14 (66.7%)	13 (86.7%)	^§^ 0.252
	Yes	7 (33.3%)	2 (13.3%)	
Congenital Heart Disease	No	15 (71.4%)	11 (73.3%)	^§^ 1.000
	Yes	6 (28.6%)	4 (26.7%)	
Delayed Toilet Training	No	8 (38.1%)	11 (73.3%)	^β^ 0.037
	Yes	13 (61.9%)	4 (26.7%)	
Inadequate Stool Consistency (Bristol 1–2)	No	19 (90.5%)	12 (80.0%)	^§^ 0.630
	Yes	2 (9.5%)	3 (20.0%)	
Age (Months)	Median (IQR)	48.9 (19.9–90.3)	33.3 (14.1–95.4)	* 0.657

Statistical test = ^β^ chi-square; ^§^ Fisher’s exact test; IQR, interquartile range. * Comparison between groups with and without constipation by the Mann–Whitney test.

## Data Availability

The data presented in this study are available on request from the corresponding author. The data are not publicly available due to ethical restrictions.

## Supplementary Materials

The following supporting information can be downloaded at https://www.mdpi.com/article/10.3390/biomedicines14010162/s1; Figure S1. Bristol scale of fecal consistency. Figure S2. Flowchart of the investigation of bowel habits and follow-up of children with Down syndrome (DS) by pediatricians. FC, functional constipation; GIT, gastrointestinal tract.
