# Supplementary material for: Prevalence of Functional Constipation in Children with Down Syndrome: A Study Conducted at a General Pediatrics Service
Source: Biomedicines. 2026 Jan 12;14(1):162. doi: 10.3390/biomedicines14010162 (PMC12838695; doi:10.3390/biomedicines14010162)

SUPPLEMENTARY FILES

Figure S1. Bristol scale of fecal consistency

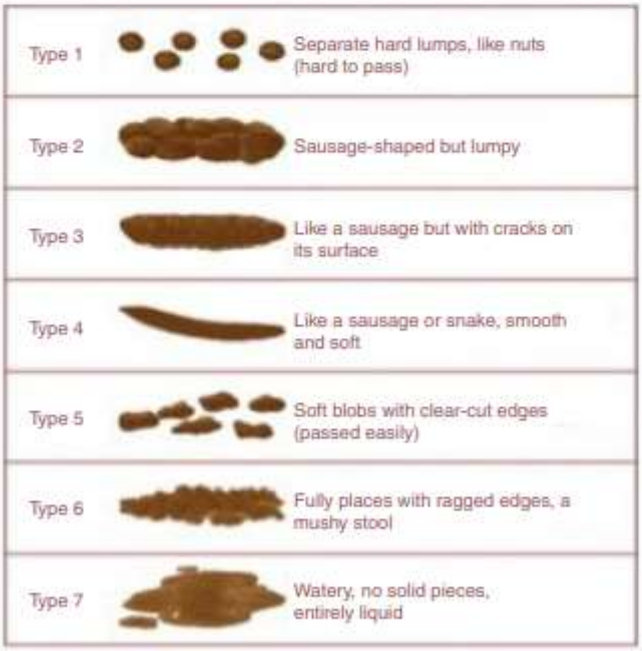

Fig. 1 – Bristol scale of fecal consistency.

Figure S2. Flowchart of the investigation of bowel habits and follow-up of children with Down syndrome (DS) by pediatricians. FC, functional constipation; GIT, gastrointestinal tract.

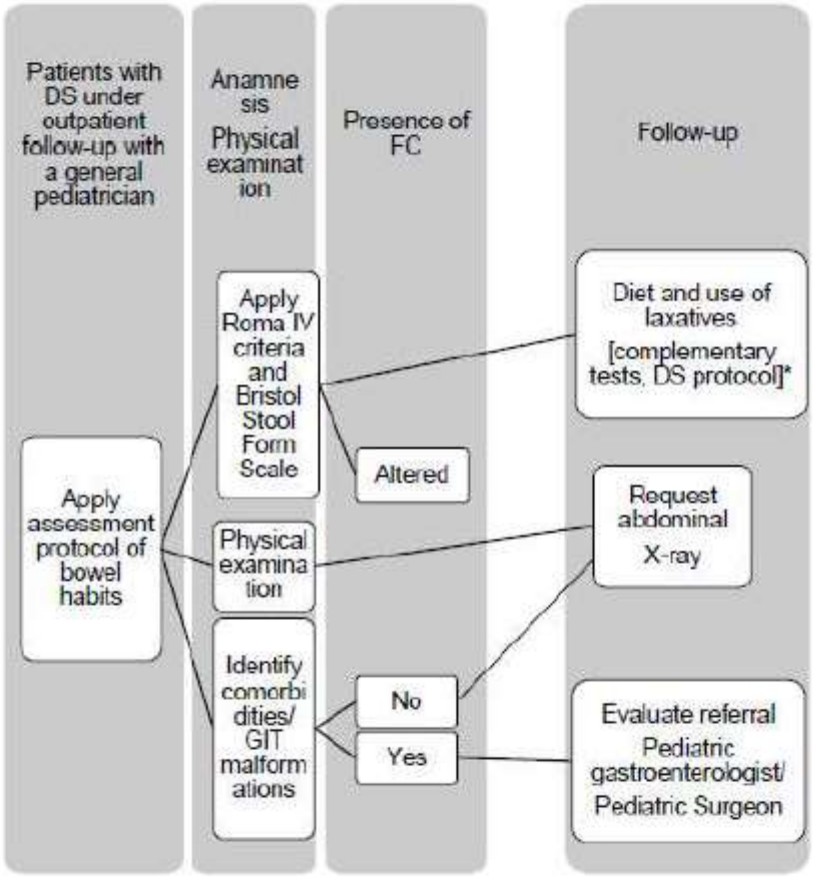

Supplement: Supplementary file 1 [file biomedicines-14-00162-s001.zip › biomedicines-3921981-supplementary.pdf]
